# Genome sequencing of correlated pathogenic *Escherichia coli* and *Enterococcus* associated with recurrent urinary tract infections

**DOI:** 10.1128/mra.00147-24

**Published:** 2024-06-25

**Authors:** Jacob Hogins, Hung P. Do, Marjan Ganjali Dashti, Philippe E. Zimmern, Larry Reitzer

**Affiliations:** 1 Department of Molecular and Cellular Biology, The University of Texas at Dallas, Richardson, Texas, USA; 2 Department of Urology, The University of Texas Southwestern, Dallas, Texas, USA; Loyola University Chicago, Chicago, Illinois, USA

**Keywords:** urinary tract infection, *Escherichia coli*, *Enterococcus*, pathogenesis, pathogens

## Abstract

Reported here are the sequences for 11 *Escherichia coli* and four *Enterococcus* strains isolated from post-menopausal women with a recurrent urinary tract infection. Each of the *Enterococcus* strains were isolated along with an *E. coli* strain. This provides a resource of high-quality complete genomes from polymicrobial infections.

## ANNOUNCEMENT

Urinary tract infections can result from *Escherichia coli* with or without a coinfecting *Enterococcus faecalis*. Coinfections with *E. faecalis*, which suppresses innate immunity, have worse outcomes (1, 2). We present genome sequences for four paired *E. coli-Enterococcus* strains and six *E. coli* strains from single-species infections.

Urine samples were collected (IRB study STU 032016-006) from women aged 36 to 81 (67 average) as described (3). *Enterococcus* strains (metallic blue colonies on chrome agar) were separated from *E. coli* using bile esculin azide agar, then outgrown in brain heart infusion (BHI) and frozen. Paired *E. coli-Enterococcus* strains were named KE and HRH, respectively.

For low molecular weight DNA isolation, revived cells were plated on chromogenic agar, inoculated into 1 mL LB (*E. coli*) or BHI (*Enterococcus*) and grown for 4 h at 37°C. Ampicillin (300 µg/mL) and chloramphenicol (50 µg/mL) were added 30–60 min before cell collection. *E. coli* DNA extraction followed the Qiagen blood and tissue kit (QBTK) instructions. *E. faecalis* DNA isolation, adapted from reference (4), involved cell pellet resuspension in 180 µL lysis buffer with 250 U mutanolysin, 20 mg/mL lysozyme, and 7.5 mg/mL RNase A; 1.5-h incubation at 37°C in a shaking heat block (1,000 rpm); addition of 200 µL buffer AL and 20 µL proteinase K (QBTK); and 30-min incubation at 56°C. QBTK instructions were then followed from the ethanol addition step. High molecular weight DNA extraction followed the same procedures except for growth in 5 mL medium, harvest at ~OD_600_ 10, addition of 250 U mutanolysin to the enzymatic lysis step for the *E. coli* extraction, and isolation using Qiagen genomic tips.

SeqCenter (Pittsburgh, PA) performed hybrid sequencing. Illumina sequencing libraries were prepared using the tagmentation-based and PCR-based Illumina DNA Prep kit and custom IDT 10 bp unique dual indices with a target insert size of 320 bp. Illumina sequencing was performed on an Illumina NovaSeq 6000 sequencer producing 2 × 151 bp paired-end reads. Demultiplexing, quality control, and adapter trimming were performed with bcl-convert (v4.1.5).

Sample libraries were prepared using the PCR-free Oxford Nanopore Technologies (ONT) Ligation Sequencing Kit (SQK-NBD114.24) with the NEBNext Companion Module (E7180L) to the manufacturer’s specifications. Nanopore sequencing was performed on an Oxford Nanopore a MinION Mk1B sequencer or a GridION sequencer using R10.4.1 flow cells. The 400 bps sequencing mode was used with a minimum read length of 200 bp. Adaptive sampling was not enabled. Guppy (v6.4.6) was used for super-accurate basecalling, demultiplexing, and adapter removal.

Porechop (0.2.4 default parameters) (5) trimmed residual adapter sequence from the ONT reads. *De novo* genome assemblies were generated from ONT read data with Flye (2.9.2 –asm-coverage 50 –genome-size 6000000) (6) under the nano-hq (ONT high-quality reads) model. Pilon (1.24 default parameters) (7) polished the assembly using Illumina read data with default parameters. Long-read contigs with an average short-read coverage of 15× or less were removed from the assembly. After assembly, contigs were evaluated for circularization via circulator (1.5.5) (8) using the ONT long reads, annotated using PGAP (6.6) (9), and statistics recorded with QUAST (5.2.0 default parameters) (10).

**TABLE 1:** Relevant parameters for assembly and access of the strains described

Assembly	BioSample	SRA accessionno.	GenBankaccession no.	Total no. of raw nanopore reads	Total no. of trimmed nanopore reads	Total no. of Illumina reads (R1 + R2)	No. of contigs	Largest contig size (bp)	Total length (bp)	Coverage of total genome (×)	Chromosome/plasmid	GC content (%)	N_50_ Nanopore reads (bp)	No. of predicted genes
KE40	SAMN38673171	SRS19762639	GCA_034644455.1	104,704	104,349	7,817,170	1	5,154,153	5,154,153	108	Chromosome	50.75	22,942	5,232
							1		151,962		Plasmid	52		187
HRH40	SAMN38673172	SRS19762640	GCA_034644395.1	69,355	69,049	7,345,398	1	2,944,957	2,944,957	101	Chromosome	37.21	14,560	2,851
							1		57,805		Plasmid	34.4		73
							1		19,762		Plasmid	32		21
							1		97,011		Plasmid	32.8		116
KE41	SAMN38673173	SRS19762644	GCA_034644215.1	93,765	93,559	9,029,832	1	5,050,178	5,050,178	106	Chromosome	50.7	24,138	5,113
							1		72,337		Plasmid	53		84
							1		101,664		Plasmid	52.6		112
HRH41	SAMN38673174	SRS19762645	GCA_034644175.1	98,810	98,384	11,803,764	1	2,619,216	74,657	210	Plasmid	33.9	20,750	90
							1		2,619,216		Chromosome	38.1		2,599
KE46	SAMN38673175	SRS19762646	GCA_034643955.1	71,324	71,003	9,749,544	1	4,736,141	4,736,141	101	Chromosome	50.71	26,792	4,734
HRH46	SAMN39888444	SRS20420947	GCA_036689605.1	47,953	47,832	8,448,426	1	2,975,538	2,975,538	102	Chromosome	37.4	15,022	2,952
							1		47,823		Plasmid	34.9		50
							1		64,713		Plasmid	34.4		85
KE47	SAMN38673176	SRS19762647	GCA_034643695.1	91,728	91,665	8,908,920	1	4,938,057	206,406	78	Plasmid	50.71	20,564	269
							1		4,938,057		Chromosome	50.8		4,893
KE48	SAMN38673177	SRS19762648	GCA_034643255.1	92,054	91,709	8,447,108	1	4,638,488	4,638,488	96	Chromosome	50.49	20,074	4,570
							1		385,579		Plasmid	48.6		381
							1		42,027		Plasmid	48.5		41
							1		8,274		Plasmid	47.1		7
KE50	SAMN38673178	SRS19762649	GCA_034642955.1	113,616	113,318	9,956,810	1	4,960,613	127,487	109	Plasmid	49.3	22,872	144
							1		4,960,613		Chromosome	50.7		4,940
KE51	SAMN38673179	SRS19762650	GCA_034642855.1	124,916	124,944	7,118,770	1	5,184,850	5,184,850	107	Chromosome	50.55	19,512	5,229
							1		134,237		Plasmid	52.2		163
KE54	SAMN38673180	SRS19762651	GCA_034642615.1	164,985	165,369	7,522,556	1	5,293,947	161,809	116	Plasmid	52	18,004	191
							1		5,293,947		Chromosome	50.7		5,380
KE55	SAMN38673181	SRS19762641	GCA_034641715.1	99,713	99,372	8,589,060	1	4,870,101	4,870,101	106	Chromosome	50.58	18,940	4,699
HRH55	SAMN38673182	SRS19762642	GCA_034642355.1	115,162	115,210	10,447,828	1	2,909,391	2,909,391	148	Choromsome	37.34	22,190	2,883
							1		69,873		Plasmid	33.7		81
KE58	SAMN38673183	SRS19762643	GCA_034640615.1	92,868	92,491	9,251,238	1	4,743,726	4,743,726	117	Chromosome	50.87	21,182	4,761

## Data Availability

The complete sequences were deposited in GenBank. The BioProject accession number is PRJNA1049017. BioSample number, SRA accession, and GenBank accession numbers are provided in Table 1.
